# Genome-Wide Analysis for the Regulation of Gene Alternative Splicing by DNA Methylation Level in Glioma and its Prognostic Implications

**DOI:** 10.3389/fgene.2022.799913

**Published:** 2022-03-04

**Authors:** Zeyuan Yang, Yijie He, Yongheng Wang, Lin Huang, Yaqin Tang, Yue He, Yihan Chen, Zhijie Han

**Affiliations:** ^1^ Department of Bioinformatics, School of Basic Medicine, Chongqing Medical University, Chongqing, China; ^2^ International Research Laboratory of Reproduction and Development, Chongqing Medical University, Chongqing, China; ^3^ Group of Mathematics Education Teaching and Research, Chongqing Fudan Secondary School, Chongqing, China

**Keywords:** glioma, alternative splicing, methylation modification, clinical prognosis, TCGA

## Abstract

Glioma is a primary high malignant intracranial tumor with poorly understood molecular mechanisms. Previous studies found that both DNA methylation modification and gene alternative splicing (AS) play a key role in tumorigenesis of glioma, and there is an obvious regulatory relationship between them. However, to date, no comprehensive study has been performed to analyze the influence of DNA methylation level on gene AS in glioma on a genome-wide scale. Here, we performed this study by integrating DNA methylation, gene expression, AS, disease risk methylation at position, and clinical data from 537 low-grade glioma (LGG) and glioblastoma (GBM) individuals. We first conducted a differential analysis of AS events and DNA methylation positions between LGG and GBM subjects, respectively. Then, we evaluated the influence of differential methylation positions on differential AS events. Further, Fisher’s exact test was used to verify our findings and identify potential key genes in glioma. Finally, we performed a series of analyses to investigate influence of these genes on the clinical prognosis of glioma. In total, we identified 130 glioma-related genes whose AS significantly affected by DNA methylation level. Eleven of them play an important role in glioma prognosis. In short, these results will help to better understand the pathogenesis of glioma.

## Introduction

Glioma is the most common and highly malignant primary intracranial tumor which is characterized by substantial heterogeneity and extremely poor prognosis in central nervous system (CNS) (Dong and Cui 2020; Pan et al., 2021). The World Health Organization (WHO) defines grade IV glioma as the glioblastoma (GBM). The annual incidence of this disease worldwide is about 5 cases per 100,000 people (Hottinger et al., 2014), and shows a significant mortality and unclarified molecular mechanism of the occurrence and development (Hottinger et al., 2014; Dong and Cui 2020). Although the etiology of glioma has been extensively studied, there are still many challenges and unknowns in the epigenetic mechanism of its pathogenesis and progress (Molinaro et al., 2019).

Recently, the DNA methylation has been demonstrated to extensively participate in the epigenetic mechanisms of CNS (Hwang et al., 2017), and many methyltransferase and demethylase-related genes (e.g., MGMT, CD44, HYAL2, SPP1, MMP2) contribute to the pathogenesis of glioma (Weller et al., 2010; Wiestler et al., 2014; Xiao et al., 2020). A large amount of the evidence showed that DNA methylation is involved in the occurrence and development of glioma tumors (Etcheverry et al., 2010; Chen et al., 2020; Dong and Cui 2020). For example, in GBM patients, the disease-related important signaling pathways (e.g., RB1 and TP53) are affected by CpG island promoter hyper-methylation (Etcheverry et al., 2010). The promoter methylation of DNA repair enzymes (O6-methylguanine-DNA methyltransferase) has been identified as a significant prognostic factor for temozolomide resistance in GBM patients (Chen et al., 2020).

Conversely, the previous studies reported that pathogenesis of glioma is significantly associated with the dysregulated alternative splicing (AS) in the brain (Mogilevsky et al., 2018; Pattwell et al., 2020; Zeng et al., 2020). AS is the primary driving force behind generating diverse proteins, which is the basis for the remarkable and complex functional regulation seen in eukaryotic cells (Xie et al., 2019). Genome-wide studies showed that 90–95% of human genes undergo some level of AS, and almost one-third of them were proved to be generated multiple protein isoforms (Kim et al., 2014; Wang et al., 2021). These processes usually show an extreme complexity in brain tissues and can play an important role in the progression of many CNS diseases (Merkin et al., 2012; Galarza-Munoz et al., 2017; Consortium 2020). For glioma, for instance, Mogilevsky et al. discovered that the manipulation of MKNK2 AS significantly suppressed the oncogenic properties of GBM cells and resensitized the cells to chemotherapy (Mogilevsky et al., 2018). Pattwell et al. found that a truncated splice variant, TrkB.T1, increases PDGF-induced Akt and STAT3 signaling and further enhances PDGF-driven GBM *in vivo* (Pattwell et al., 2020). Moreover, many previous studies indicate that there is a strong link between DNA methylation and AS and it generally contributes to the pathogenesis of CNS disorders, including glioma (Feng et al., 2019; Li et al., 2019). For example, transcriptome analysis revealed that PTEN methylation influences mature mRNA transcripts through modulation of pre-mRNA AS, and the methylation-defective PTEN R159K mutant is found most frequently in glioma patients. There was mark dysregulation of splicing factors in the PTEN-deficient GBM samples (Feng et al., 2019). The important oncogene METTL3 is a methyltransferase and it is found to modulate the nonsense-mediated mRNA decay of splicing factors and AS isoform switches in GBM. The methylation modification of serine- and arginine-rich splicing factors by METTL3 promotes GBM tumor growth and progression (Li et al., 2019).

**FIGURE 1:**
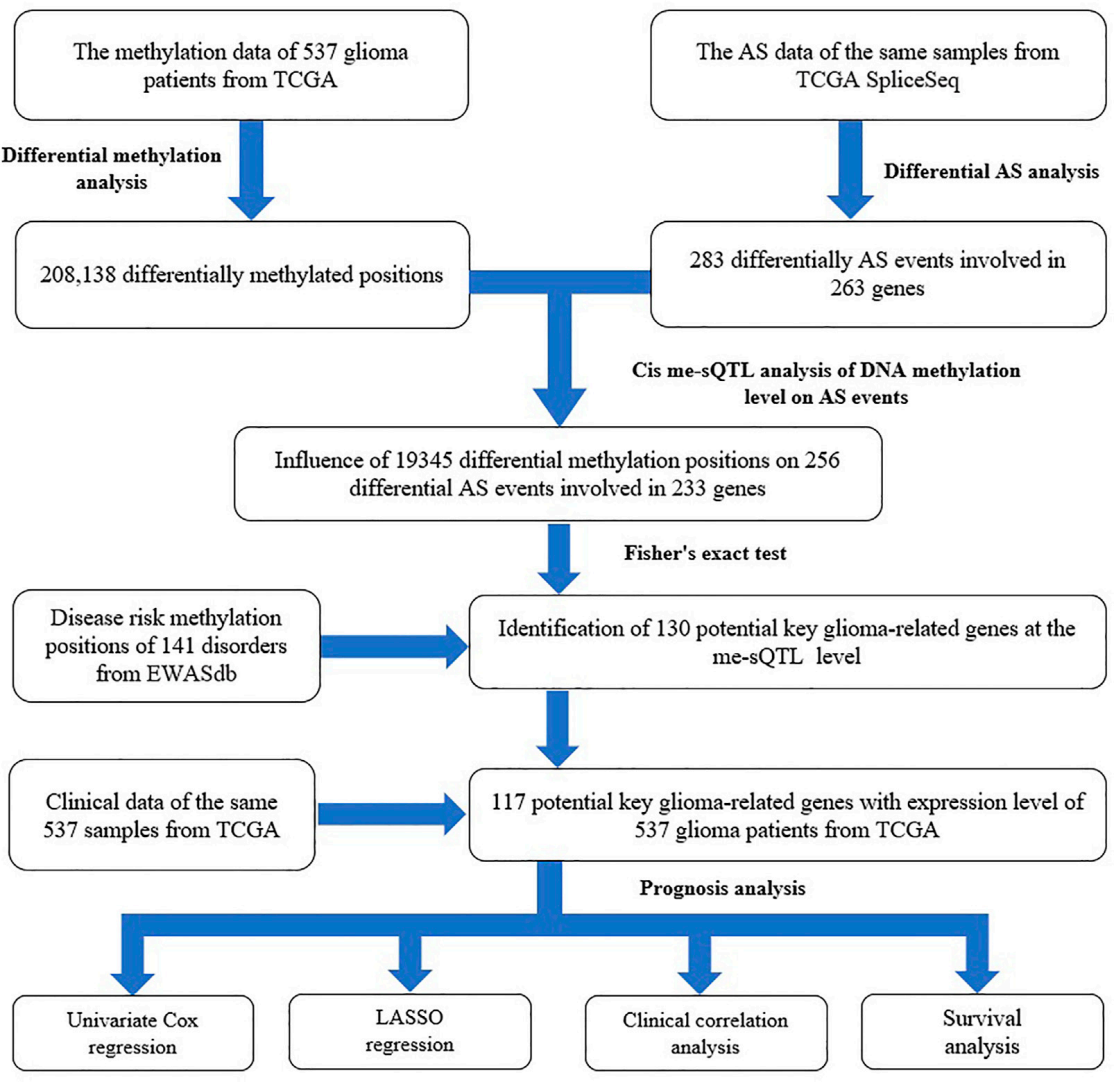
The flow chart of the study design for exploring the influence of DNA methylation level on gene AS in glioma and its impact on disease prognosis.

However, so far, there has been no systematic study to explore the relationship between glioma-related DNA methylation and gene AS in the whole genome scale, and the influence of their interaction on the pathogenesis and progress of glioma. Therefore, in this study, we performed a genome-wide analysis by integrating the DNA methylation and AS data of 537 low-grade glioma (LGG) and GBM individuals. First, we downloaded the relevant data from the Cancer Genome Atlas (TCGA), TCGA SpliceSeq and EWASdb database, respectively. Second, we conducted the differential analysis between LGG and GBM samples to identify the glioma-related methylation positions and AS events. Third, based on the results, we performed a splicing quantitative trait methylation loci (defined as me-sQTL (Gutierrez-Arcelus et al., 2015; Han and Lee 2017)) analysis to explore the influence of DNA methylation level on gene AS in glioma. Fourth, we further explored the characteristics of these me-sQTLs and affected AS events. Fifth, combining the data of disease risk methylation positions from EWASdb, we performed the two-tailed Fisher’s exact test to investigate the disease specificity of the me-sQTLs and identify the potential key genes related to them in glioma. Finally, based on these potential key genes and clinical data, we conducted the least absolute shrinkage, univariate Cox regression, selection operator (LASSO) regression, clinical correlation and survival analysis to explore the influence of these genes whose AS events affected by DNA methylation on clinical prognosis of glioma. The flow chart is shown in Figure 1.

## Materials and Methods

### Data Collection and Processing

Clinical and methylation information of glioma patients was downloaded from the TCGA database (http://cancergenome.nih.gov), a comprehensive resource containing multi-omics data from various cancers. According to the annotation of TCGA, glioma is classified as the LGG and the GBM. TCGA is a global genomic profiling project that utilizes high-throughput microarray technologies to identify molecular subtype classifications of cancers, multigene clinical predictors, new targets for drug therapy, and predictive markers for these therapies (Vigneswaran et al., 2015). The International Classification of Diseases for Oncology has been used for nearly 25 years as a tool for coding diagnoses of neoplasms in tumor and cancer registrars and in pathology laboratories (Warzel et al., 2003). Data analysis was performed with the glioma classification LGG and GBM provided by the TCGA database. Current glioma classifications are based on the 2007 WHO grading scale, which separates gliomas based on cytologic features and degrees of malignancy after hematoxylin and eosin (H&E) staining (Erridge et al., 2011). According to the classification of gliomas in the TCGA database, data analysis is carried out by using the classifications LGG and GBM of gliomas provided by the TCGA database. We accessed these TCGA data using the Genomic Data Commons (GDC) data portal (https://portal. gdc. cancer.gov/). Particularly, based on our previous study (He et al., 2020), we first selected “DNA methylation” for the Data Category, “Illumina human methylation 450” for the Platform, “brain” for the Primary Site and “gliomas” in the Disease Type to screen out the suitable methylation array of patients in the GDC data portal. Then, the“clinical,” “brain” and “gliomas” were selected to the Data Category, Primary Site and Disease Type, respectively, to screen out the clinical information of patients in the GDC data portal. Finally, we removed samples that lacked methylation or clinical information.

The AS events of these samples were obtained from the TCGA SpliceSeq database (http://bioinformatics.mdanderson.org/TCGASpliceSeq), which identifies AS events and describes their genome profiles using the RNA-seq data of the TCGA samples (Ryan et al., 2016). Particularly, we downloaded the AS isoform average percent spliced-in (PSI) values of the LGG and GBM samples, respectively, from TCGA SpliceSeq database with the common parameter settings (i.e., the percentage of samples with PSI value >75%, minimum PSI range >0 and minimum PSI standard deviation >0.1) according to the previous studies (Yang et al., 2019; Rong et al., 2020; Wei et al., 2021). Based on the classification criteria of TCGA SpliceSeq, we classified the types of AS events into Alternate Acceptors (AA), Alternate Donors (AD), Exon Skip (ES), Retained Intron (RI), Alternate Promoters (AP), Alternate Terminators (AT) and Mutually Exclusive Exons (ME). The AS events that are not present in both LGG and GBM samples were removed.

Moreover, the information of disease risk methylation positions was obtained from the EWASdb database (http://www.bioapp.org/ewasdb/index.php/Index/index). EWASdb is a specialized epigenome-wide association database which stores the results of 1,319 epigenome-wide association study (EWAS) studies involved in the 302 diseases/phenotypes with the threshold for significance *p* < 1 × 10^–7^ (Liu et al., 2019). We downloaded the EWAS single epi-marker and annotation files (phenotype/disease info) and merged the files by the disease codes.

### Differential Analysis of Methylation Positions

To obtain the glioma-related methylation positions, we performed differential methylation analysis between GBM and LGG samples. In particular, we used a Subset-quantile Within Array Normalization method to preprocess the methylation data by the R package “minfi,”, a specialized tool for the analysis of the Illumina methylation 450 array dataset (http://bioconductor.org/packages/release/bioc/html/minfi.html) (Aryee et al., 2014). Then, the quality control of methylation array was conducted “densityBeanPlot” function of this package. The characteristics of the qualified samples show that the methylation levels (beta values) of CpG positions are distributed around 0 and 1, respectively. Finally, based on the qualified methylation array data, we used a bump-hunting algorithm to identify the differentially methylated positions between GBM and LGG subjects by the “dmpFinder” function of this package. The parameter was set by its default value (i.e., type = “categorical”) and the significance level was set according to a common threshold for the absolute intercept ≥0.2 (i.e. 20% difference on the beta values) and the *p* value <1 × 10^–3^ (Guo et al., 2015).

### Differential Analysis of Alternative Splicing Events and Annotation

To identify the glioma-related AS events and corresponding genes, we performed the differential AS events analysis and gene annotation. Particularly, the differential AS events analysis was conducted by the vast-tools software (Irimia et al., 2014). Based on the PSI of each AS event, we performed a Bayesian inference-based differential AS analysis by the “diff” function of vast-tools software with its default parameters. According to the previous studies, we set the threshold for significance at the minimum value for absolute value of differential PSI between GBM and LGG samples (MV|ΔPSI|) at 0.95 confidence level greater than 10% (Ha et al., 2021; Hekman et al., 2021). The gene annotation was conducted by g:Profiler toolset, a web server for conversions between gene identifiers and functional annotation (Raudvere et al., 2019). We used the g:Profiler to identify these AS events corresponding genes, convert their ID and annotate the genome location and type of the genes. The annotation file (hg19) from the database (release 75) were used for these analyses (Aken et al., 2017).

### Association Analysis Between DNA Methylation and Alternative Splicing

To explore the effect of methylation on AS events in glioma, we performed a cis me-sQTL analysis by combining the PSI values of differential AS events and the beta values of differentially methylated positions from the same samples. Particularly, we first considered the distance between the differentially methylated positions and the transcription initiation site (TSS) of differential AS events corresponding genes less than 1 M as the cis region, and selected all methylation positions and AS event pairs that met the conditions for the cis me-sQTL analysis. The annotation files of the Illumina methylation 450 array dataset (hg19) and Ensembl database (release 75) were used to locate the genomic locations of the methylated positions and the TSS of AS events corresponding genes, respectively. Then, based on the beta values of the differentially methylated positions in combination with the PSI values of the corresponding differential AS events, we used a linear regression model to perform a cis me-sQTL analysis by the R package “Matrix eQTL” with the parameters, age, and gender as covariates (Shabalin 2012). Finally, we conducted a multiple testing by Benjamini–Hochberg method to correct the *p* values of the cis me-sQTL analysis and set false discovery rate (FDR) q value less than 0.05 as the threshold for significance level according to the previous studies (Gillies et al., 2018; Drag et al., 2019; Han et al., 2020).

### Disease Specificity Analysis of the Cis Me-sQTLs

In order to explore the disease specificity of these cis me-sQTLs and further verify our findings as well as identify the potential key glioma-related genes with affected AS events by methylation level, we performed the two-tailed Fisher’s exact test by combining the disease risk methylation positions and the results of cis me-sQTLs analysis. Particularly, we first produced the disease risk methylation position datasets for various disorders including glioma from EWASdb database (Liu et al., 2019). Then, we defined the methylation positions which were unlikely to have an effect on the AS events in cis region (*p* > 0.05) as the non me-sQTLs. Next, by the two-tailed Fisher’s exact test, we compared the proportions of all these cis and non me-sQTLs in the disease risk methylation positions for each of the disorders to explore the disease specificity and further verify previous findings. The threshold for significance level was set as the *p* value <0.05. Finally, to identify the potential key glioma-related genes at the me-sQTL level, we compared the proportions of cis and non me-sQTLs in glioma-related methylation positions for each gene using the two-tailed Fisher’s exact test (the threshold of *p* < 0.05). The “fisher.test” function of R was used for these calculations.

### Influence of the Me-sQTL Genes on Clinical Prognosis of Glioma

We further analyzed the influence of these potential key genes whose AS events are affected by DNA methylation on clinical prognosis of glioma. First, we calculated the average expression of these genes in each individual and separated the samples into low and high expression groups according to the median of average expression. Then, we used the Kaplan-Meier overall survival curves to compare prognosis between the high expression and low expression individuals. Next, we performed a univariate Cox regression analysis to assess the association between these me-sQTL genes and the prognosis of glioma. The threshold of significance was set at 95% confidence interval (CI) of hazard ratio (HR) ⊉ 1 and *p* < 0.05. Then, based on the results of univariate Cox regression analysis, the R package “glmnet” was used to perform the LASSO regression analysis, a fit algorithm based on cyclical coordinate descent and warm start search along a regularization path, to identify the main glioma prognosis-related genes (Simon et al., 2011). According to the common parameter settings, the maxit and alpha were set at 1,000 and 1, respectively, and others were set by their default values. Based on the results, the risk scores were calculated for each subject by the R package “survival” (http://CRAN.R-project.org/package=survival). Further, the receiver operator characteristic (ROC) curve was used to verify the reliability of these risk scores by the R package “survivalROC” (https://CRAN.R-project.org/package=survivalROC). Finally, we used the chi-square test to assess the association between the expression of these glioma prognosis-related genes and other clinical features of the patients, which included the age at initial pathologic diagnosis, the vital status, and the gender. The threshold for significance was set at the *p* value <0.05.

## Results and Discussion

### The Multi-Omics Data From 537 Glioma Individuals

**FIGURE 2:**
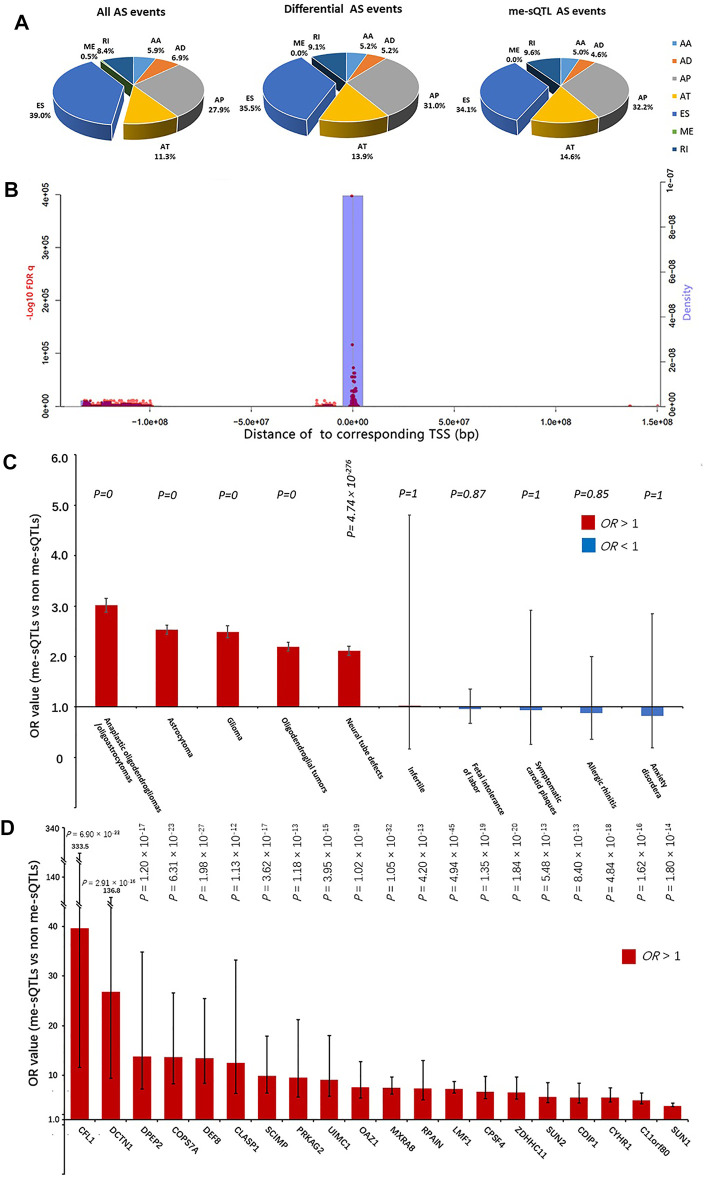
The characteristic of the cis me-sQTLs and the affected AS events. **(A)** The pie charts show the proportion in all (left), differential (middle) and DNA methylation affected AS events (right) annotated with each class (AA, AD, ES, RI, AP, AT and ME), respectively. **(B)** The blue bar graphs indicate the relationship between the abundance of the cis me-sQTLs and the distance of them to TSS of corresponding AS events. The red dots indicate the relationship between the statistical significance of the cis me-sQTLs associated with AS and the distance of them to TSS of corresponding AS events. **(C)** The disease specificity of the cis me-sQTLs by the two-tailed Fisher’s exact test. **(D)** The glioma specificity of the cis me-sQTLs in each gene by the two-tailed Fisher’s exact test. The black bars in histogram represent 95% confidence intervals.

In total, we obtained the datasets of DNA methylation values, AS events PSI values, gene expression levels and clinical information from 537 glioma samples (including 486 LGG and 51 GBM patients). The summary of these glioma samples was shown in Table 1. Particularly, after the missing value filtering and normalization processing, we quantified a total of 369,531 CpGs methylation positions with the normalized values according to the annotation files of Illumina human methylation 450 array. The results of normalization processing were shown in the Supplementary Figure S1 and our previous study (He et al., 2020). We obtained 7,414 AS events with the PSI values from 537 glioma samples the TCGA SpliceSeq database. These AS events are composed of about 39.0% ES, 27.8% AP, 11.3% AT, 8.4% RI, 6.9% AD, 5.9% AA and 0.5% ME types (Figure 2A). The expression data of 20,530 genes of the glioma samples was downloaded from the TCGA database and quantified by RSEM values. The clinical information of these samples contains age, gender, survival time, and vital status. Moreover, after the combination of same disease types and missing value filtering, we obtained a total of 141 disease risk methylation position data sets from the EWASdb database.

**TABLE 1:** Summary of the 537 individuals studied in this work.

Individuals	Sample Type	Sample Size	Mean Age (Aken et al.)	Male/Female (Han and Lee)	Death Rates (Han and Lee)
GBM subjucts	Primary Tumor	51	61.54 (13.41)	56.00/44.00	66.00
LGG subjucts	Primary Tumor	486	42.91 (13.42)	54.64/45.36	25.15
Total		537	44.66 (14.48)	54.77/45.23	28.97

These samples are from our previous study (He et al., 2020).

### Differential Analysis of Methylation Positions and Alternative Splicing Events

We performed a differential methylation analysis between the LGG and GBM subjects to identify the glioma-related DNA methylation positions. All of the methylation array data met quality control metrics. The results showed that the beta values of DNA methylation positions are mainly distributed around 0 and 1, respectively, for each sample. The details are described in the Supplementary Figure S2 and our previous study (He et al., 2020). By the differential methylation analysis, we identified a total of 208,138 positions with a significantly different methylation level between LGG and GBM subjects. The results are shown in the Supplementary Table S1 and our previous study (He et al., 2020).

**FIGURE 3:**
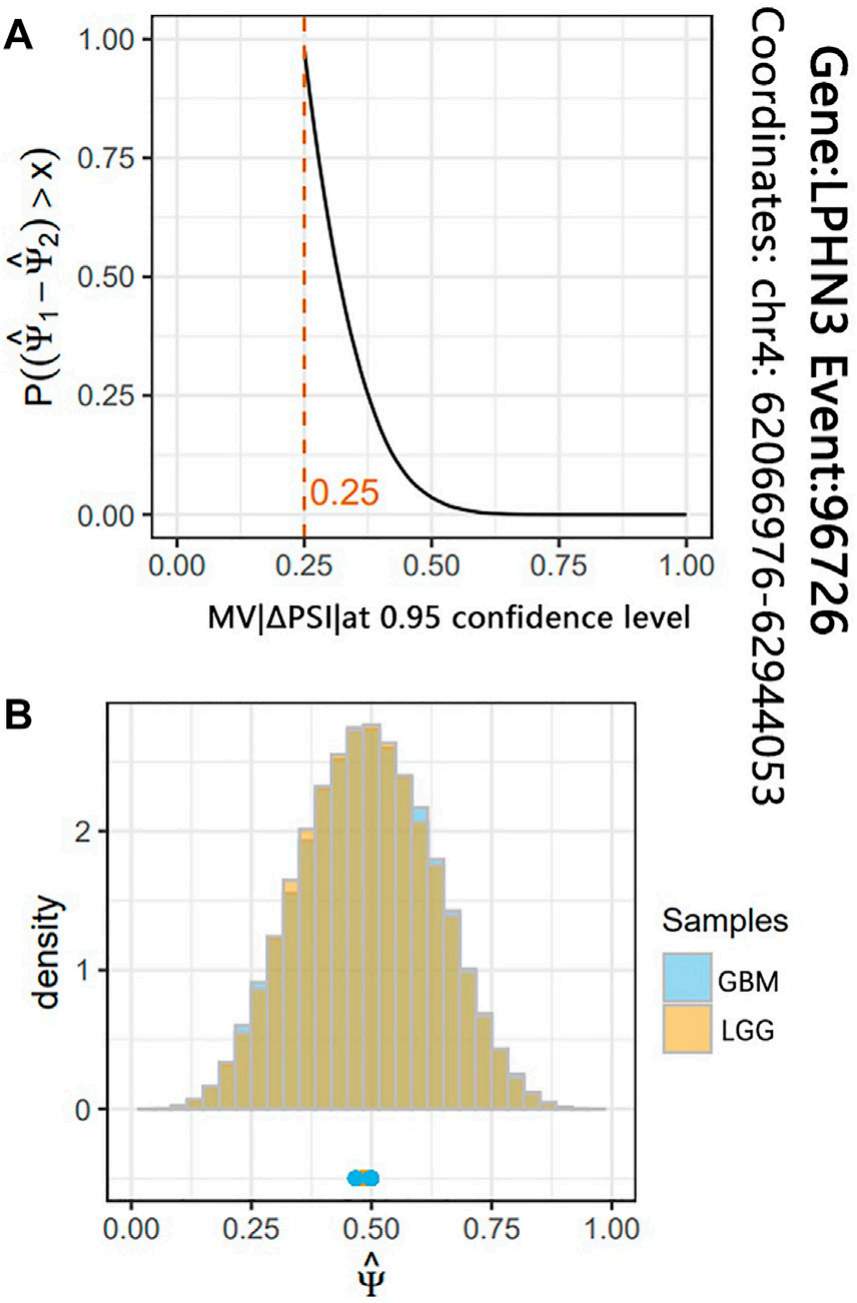
The results of differential analysis for the AS event 96726 of LPHN3 gene. **(A)** The red line indicates that the maximum probability of ΔPSI of AS event 96726 between LGG and GBM subjects is greater than 0.25. **(B)** The histogram shows the two joint posterior distributions over PSI and the point estimates for each replicate.

To identify the glioma-related AS events, we further conducted differential AS events between the LGG and GBM subjects. According to the significance threshold MV|ΔPSI| at 0.95 confidence level ≥10%, we identified a total of 287 differential AS events between LGG and GBM subjects. These differential AS events belonged to 263 genes (Supplementary Table S2). Figure 3 shows the most significant differential AS events (SpliceSeq ID: 96726) of LPHN3 gene (MV|ΔPSI| at 0.95 confidence level = 0.25). A recent study reported that LPHN3 was an important paralog of EVA1C which leads to the high infiltration levels of multiple immune cells in glioma (Hu and Qu 2021). Moreover, according to the classification criteria for SpliceSeq database, about 35.5%, 31.0%, 14.0%, 9.1%, 5.2% and 5.2% of these identified AS events are categorized into ES, AP, AT, RI, AD and AA types, respectively (Figure 2A). We did not find a significant difference in the proportion of AS event types when compared with the original AS event type proportion by the two-tailed Fisher’s exact test (Figure 2A). This revealed a typological universality of the differential AS events in glioma.

### Association Analysis Between DNA Methylation and Alternative Splicing

Combining the PSI values of differential AS events with the beta values of differentially methylated positions from the same samples, we used a linear regression model to perform the cis me-sQTL analysis by R package “Matrix eQTL” with the parameters, age, and gender serving as covariates. In total, we identified 19,345 methylated positions affecting 256 AS events which are involved in 233 genes (over 88% of the total differential genes) with a significance level of FDR *q* < 0.05. This revealed a general influence of DNA methylation level on gene AS in glioma. The top 25 significant results are shown in Table 2 (the full information is listed in the Supplementary Table S3). Among the 256 affected AS events, we found that about 34.1%, 32.2%, 14.6%, 9.6%, 4.6% and 5.0% of these affected AS events are categorized into ES, AP, AT, RI, AD and AA types, respectively. By the two-tailed Fisher’s exact test, we also did not find a significant difference of percentage between the affected and the original AS event types (Figure 2A). This revealed a typological non-specific regulation of gene AS by DNA methylation level in glioma. Further, we explored the relationship between the significance of regulation of the cis me-sQTLs and the distance of them to the TSS of the corresponding affected gene, and their distribution characteristics in genome. The results showed that these cis me-sQTLs tended to be distributed in the proximity of the corresponding affected gene TSS, and there were more significant regulatory effects of them in these regions (Figure 2B). This was consistent with the findings of previous studies (Pangeni et al., 2018; Chen and Elnitski 2019).

**TABLE 2:** The top 25 significant results of the me-sQTLs and the differential AS events affected by the methylated position.

Methylated position	Differential analysis of methylated positions				AS event	Differential analysis of AS			Me-sQTLs			
	Strand	Intercept	f	*p* Value		Gene	E (ΔPSI)	95% MV|ΔPSI|	Statistic	*p* Value	FDR	Beta
cg04928129	1429051−	−2.1144	230.3921	3.34E-44	33029	LMF1	−0.004168	0.11	28.4852	4.34E-109	9.97E-106	0.7302
cg00583426	1209990−	−2.7769	210.9880	4.05E-41	33029	LMF1	−0.004168	0.11	28.1495	1.93E-107	2.22E-104	0.6155
cg08259514	1131634−	−3.8941	291.6219	1.79E-53	33029	LMF1	−0.004168	0.11	27.7357	2.11E-105	1.61E-102	0.5842
cg04603812	1429265−	−4.5946	291.2726	2.01E-53	33029	LMF1	−0.004168	0.11	27.3596	1.51E-103	8.70E-101	0.7149
cg03323597	1131489−	−2.1121	273.3760	8.81E-51	33029	LMF1	−0.004168	0.11	26.8338	6.07E-101	2.79E-98	0.7754
cg09249980	1213919−	1.1469	137.6579	9.86E-29	33029	LMF1	−0.004168	0.11	25.2485	4.74E-93	1.36E-90	1.0011
cg00611495	1120275−	−1.0380	165.5488	1.38E-33	33029	LMF1	−0.004168	0.11	25.1518	1.44E-92	3.31E-90	0.8511
cg20104307	778658+	−1.9372	165.0035	1.71E-33	33029	LMF1	−0.004168	0.11	25.0200	6.57E-92	1.37E-89	0.7347
cg27040104	1384722−	−0.7004	129.0667	3.38E-27	33029	LMF1	−0.004168	0.11	24.8244	6.25E-91	1.20E-88	0.8274
cg00525011	122031+	−1.6582	190.3615	9.36E-38	33029	LMF1	−0.004168	0.11	24.6293	5.92E-90	1.05E-87	0.6617
cg04913730	1121907−	−1.7877	137.0784	1.25E-28	33029	LMF1	−0.004168	0.11	24.5070	2.42E-89	3.98E-87	0.6183
cg00675160	1208531+	−0.7381	141.6593	1.93E-29	33029	LMF1	−0.004168	0.11	24.4083	7.57E-89	1.16E-86	0.7337
cg08438529	1052939−	−1.2133	173.4449	6.27E-35	33029	LMF1	−0.004168	0.11	24.1224	2.05E-87	2.94E-85	0.5957
cg07549278	1204244−	−2.0317	95.9829	4.18E-21	33029	LMF1	−0.004168	0.11	23.9448	1.59E-86	2.15E-84	0.6040
cg16383109	126451−	−1.4219	232.0717	1.82E-44	33029	LMF1	−0.004168	0.11	22.9002	2.77E-81	3.35E-79	0.6947
cg05245533	795877−	−0.9085	145.6310	3.86E-30	33029	LMF1	−0.004168	0.11	22.8553	4.65E-81	5.34E-79	0.6500
cg16443148	776667−	−0.2492	47.2131	1.61E-11	33029	LMF1	−0.004168	0.11	22.6322	6.12E-80	6.12E-78	0.6401
cg09786479	1020419+	−3.2149	77.1741	1.68E-17	33029	LMF1	−0.004168	0.11	22.6067	8.22E-80	7.87E-78	0.5584
cg07336438	1131466−	−0.9484	173.4777	6.19E-35	33029	LMF1	−0.004168	0.11	22.5742	1.20E-79	1.10E-77	0.7216
cg10163825	776685+	−0.4881	18.5564	1.93E-05	33029	LMF1	−0.004168	0.11	22.5302	1.99E-79	1.76E-77	0.9054
cg27127090	1131327+	0.3781	81.8560	2.08E-18	33029	LMF1	−0.004168	0.11	22.1160	2.38E-77	1.95E-75	0.9443
cg07915516	377344−	−1.5503	116.9595	5.29E-25	33029	LMF1	−0.004168	0.11	21.8766	3.77E-76	2.99E-74	0.7060
cg06587435	865125+	1.6381	82.6033	1.49E-18	33029	LMF1	−0.004168	0.11	21.7725	1.25E-75	9.00E-74	1.1040
cg08641445	1080637+	0.4693	58.8931	6.88E-14	33029	LMF1	−0.004168	0.11	21.6790	3.68E-75	2.57E-73	0.9575
cg05272807	1232363+	0.2547	93.9919	9.94E-21	33029	LMF1	−0.004168	0.11	21.6061	8.55E-75	5.78E-73	0.7974

### Disease Specificity Analysis of the Cis Me-sQTLs

To explore the disease specificity of these cis me-sQTLs and verify our findings, as well as further identify the potential key glioma-related genes at the me-sQTL level, we performed the two-tailed Fisher’s exact test using the disease risk methylation position data from the EWASdb database. We found that the risk methylation positions of all the 141 diseases are overlapped with the cis me-sQTLs and non me-sQTLs. By comparing the proportions of cis me-sQTLs and non me-sQTLs in each disease risk methylation position dataset (the threshold of Fisher’s *p* value <0.05), we found that the cis me-sQTLs significantly enriched the risk methylation position dataset of 103 diseases, which are mainly composed of CNS disorders and malignant tumor diseases including glioma (odds ratio (OR) = 2.49, *p* = 0). In contrast, the remaining 38 diseases, whose risk methylation positions are not significantly enriched by the cis me-sQTLs, are mainly composed of the other types of disorders, e.g., the rheumatic heart disease (*p* = 5.99 × 10^–1^), septicemia (*p* = 6.97 × 10^–1^), and Infertile (*p* = 1). The top 5 most and least significant results are shown in Figure 2C (the full information is listed in the Supplementary Table S4). The results revealed the specificity and similarity of neuro-oncological disorders at the me-sQTL level and verified the association of the cis me-sQTLs we identified with glioma. Further, for each type of AS event and each gene, we compared the proportions of their cis and non me-sQTLs in glioma risk methylation position dataset, respectively. The results showed that the cis me-sQTLs of almost all types of AS events are significantly enriched in glioma risk methylation position dataset, i.e., AA (OR = 4.76, *p* = 1.18 × 10^–36^), AT (OR = 2.85, *p* = 9.63 × 10^–66^), ES (OR = 2.39, *p* = 6.26 × 10^–112^), RI (OR = 2.05, *p* = 2.85 × 10^–30^), AD (OR = 2.55, *p* = 4.78 × 10^–21^), and AP (OR = 2.88, *p* = 8.69 × 10^–191^), and there are a total of 130 genes whose cis me-sQTLs are significantly enriched in glioma risk methylation position dataset (*p* < 0.05). Figure 2D shows the top 20 significant results and full information is listed in the Supplementary Table S5. We considered that these genes are more correlated with the pathogenesis of glioma at the me-sQTL level and selected them for the following prognosis analysis of glioma.

### Influence of the Me-sQTL Genes on Clinical Prognosis of Glioma

**FIGURE 4:**
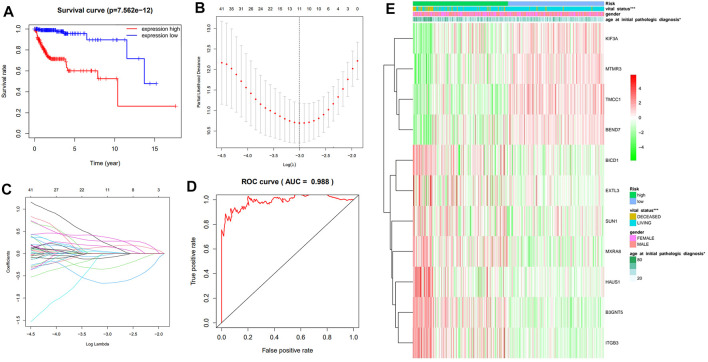
The influence of the glioma-related genes whose AS significantly affected by DNA methylation level on the disease prognosis. **(A)** The Kaplan-Meier overall survival curves of the low (red) and high (blue) expression groups. **(B)** and **(C)** show the results of LASSO regression. There are 11 independent genes with their coefficient when the partial likelihood deviance reaches its minimum value. **(D)** The ROC curve reveals the reliability of the risk score by comparing the true and false positive rate. **(E)** The heatmap shows the association between the risk scores of the prognosis-related me-sQTL genes and the clinical features of glioma patients.

We further analyzed the influence of the potential key genes which are associated with glioma in me-sQTL level on the clinical prognosis of glioma. The expression data were obtained from TCGA database and these data are involved in 117 of the 130 potential key genes. We found that the overall survival curve of the subjects with high expression of these genes is significantly longer than the subjects with low expression (*p* = 7.56 × 10^–1^ (Figure 4A). This revealed that the expression dysregulation of these potential key genes is significantly associated with the bad prognosis of glioma patients. To avoid dependence between the 117 genes and identify the main glioma prognosis-related genes, we performed the univariate Cox regression analysis of the 117 genes. However, the results showed that 61me-sQTL genes identified are high-risk factors for the prognosis of glioma subjects (i.e. 95% CI HR ⊉ 1 and *p* < 0.001) (Supplementary Figure S3). We discover that both over-expression of those 30 genes and under-expression of the other 31 genes can lead to a poor prognosis in glioma patients, which is also consistent with common sense. given that patients are in advanced stages of the disease and their survival may be affected by other complications or factors. Then, we further applied the LASSO regression algorithm to conduct the selection and calculate the risk score of each subject to univariate Cox regression results. The results showed that there are 11 genes (i.e., KIF3A, HAUS1, TMCC1, BEND7, B3GNT5, MTMR3, ITGB3, BICD1, EXTL3, SUN1 and MXRA8) identified when the cross-validated partial likelihood deviance reaches its minimum value (Figures 4B,C). Among the 11 genes, the coefficients of 7 were positive (i.e., increase risk of disease), and others were negative (i.e., decrease risk of disease). A previous study reported that the low expression of the TMCC1 gene confers poor clinical prognoses of glioma patients which is in accordance with our findings (Pangeni et al., 2018). The area under the curve (AUC) of the ROC is 0.988, which reveals the reliability of the risk score (Figure 4D). According to the median of risk scores, the patients were separated into the low and high-risk groups. We found that the LGG and GBM subjects are mainly distributed in the low and high-risk group, respectively, which reflects the consistency between the risk scores calculated by the prognosis-related me-sQTL genes and the severity of glioma. Moreover, the results of chi-square test showed that the risk scores of the prognosis-related me-sQTL genes are also associated with the age at initial pathologic diagnosis (*p* = 4.89 × 10^–2^) and vital status (*p* = 1.81 × 10^–9^), but not with the gender of the patients (*p* = 4.60 × 10^–1^) (Figure 4E). This proves that the 11 key genes we found are meaningful for clinical prognosis of glioma. Among the 11 genes, 7 of them (i.e., B3GNT5, BICD1, KIF3A, HAUS1, MTMR3, ITGB3 and EXTL3) have been confirmed to be associated with the prognosis of glioma (Kim et al., 2011; Sumazin et al., 2011; Huang et al., 2017; Zhou et al., 2018; Jeong et al., 2020; Li et al., 2020; Wang et al., 2020). Our findings imply that the functions of these genes in glioma prognosis may be related to the methylation regulation of their AS events.

## Conclusion

In this study, we used the TCGA data to explore the role of the me-sQTL process on pathogenesis of glioma and identify the affected genes and further analyze the influence of them on the clinical prognosis of glioma. In total, we identified 130 such genes which have the following three characteristics: 1) they are significantly differentially expressed between the LGG and GBM subjects; 2) their AS events are significantly regulated by DNA methylation level in the cis regions; and 3) the cis me-sQTLs of them are significantly enriched in glioma risk methylation position dataset. Further, the results of clinical data analysis show a significant association between the expression of these genes and the clinical prognosis of glioma, and among them, 11 (i.e., KIF3A, HAUS1, TMCC1, BEND7, B3GNT5, MTMR3, ITGB3, BICD1, EXTL3, SUN1 and MXRA8) are considered the key risk factors for the prognosis and severity of glioma. At the same time, these 130 genes provide new ideas for the study of the interaction between DNA methylation and alternative splicing in gliomas and similar diseases and provide reference for future research on the study of DNA methylation and variable splicing in neurological diseases in the whole genome. In summary, we performed a strategy to explore the influence of DNA methylation level on gene AS in glioma and these findings will help to better understand pathogenesis of glioma.

## Data Availability

The original contributions presented in the study are included in the article/Supplementary Material, further inquiries can be directed to the corresponding author.
