# Supplementary material for: Genome-Wide Analysis for the Regulation of Gene Alternative Splicing by DNA Methylation Level in Glioma and its Prognostic Implications
Source: Front Genet. 2022 Mar 4;13:799913. doi: 10.3389/fgene.2022.799913 (PMC8931337; doi:10.3389/fgene.2022.799913)
Supplement: Supplementary file 6 [file Image1.pdf]

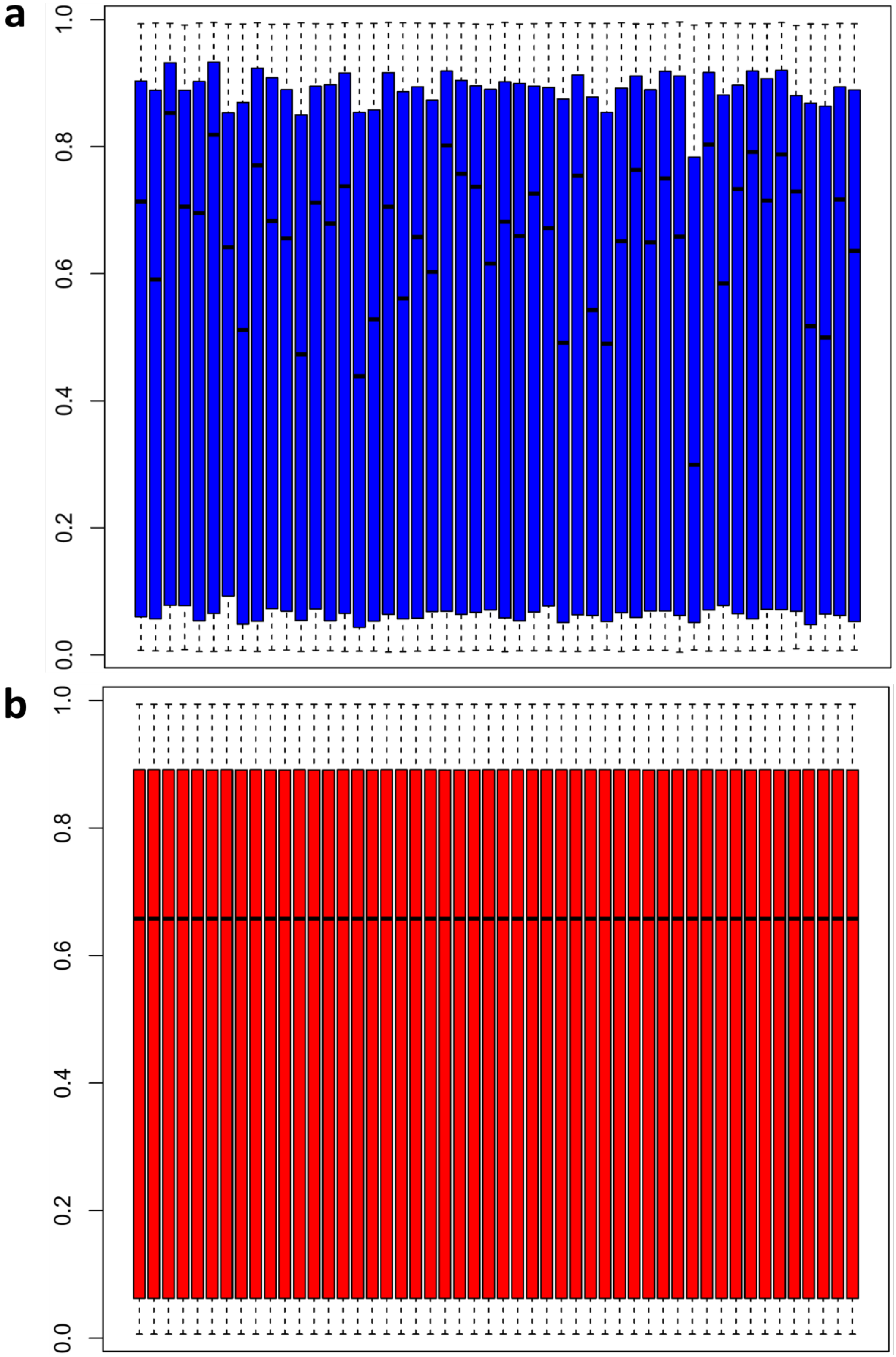

Figure S1 The results of the DNA methylation microarray data normalization. **(a)** The boxplot reveals the distribution of methylation values in 50 glioma samples selected at random without normalization processing. **(b)** The boxplot reveals the distribution of methylation values in the 50 samples after the normalization processing.

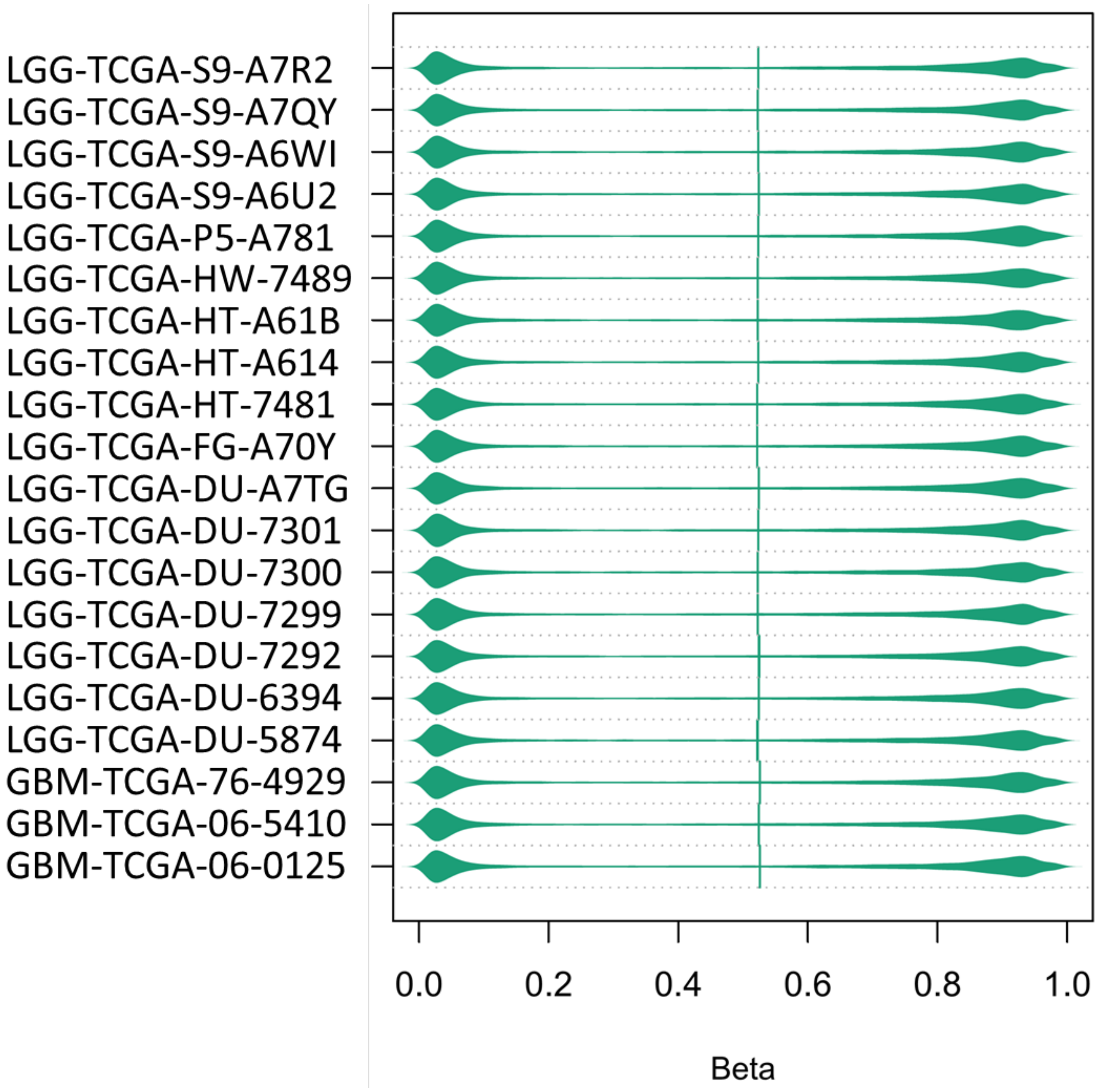

Figure S2 The results of the DNA methylation array quality control. The density bean plot reveals the distribution of the beta values of CpG methylation sites in 20 glioma samples (i.e. methylation array) selected at random.

	pvalue	Hazard ratio
ZNF707	<0.001	0.343(0.182–0.646)
PBX1	<0.001	0.549(0.408–0.738)
SUN1	<0.001	4.872(2.941–8.069)
LRRC29	0.210	0.721(0.433–1.203)
SLC39A13	0.580	1.182(0.653–2.139)
C16orf59	<0.001	1.630(1.235–2.152)
B3GNT5	<0.001	2.432(1.952–3.030)
UNC13B	<0.001	0.412(0.259–0.655)
TMCC1	<0.001	0.125(0.077–0.201)
MRPL35	0.248	0.641(0.302–1.363)
MUC20	0.038	0.801(0.651–0.987)
GPR108	<0.001	6.852(3.622–12.963)
STAMBPL1	<0.001	0.495(0.379–0.645)
ADAM15	<0.001	3.338(1.943–5.737)
CPNE1	0.187	1.363(0.861–2.158)
ZDHHC11	<0.001	0.678(0.545–0.845)
ACADVL	0.032	1.729(1.049–2.852)
ACOT8	0.018	2.288(1.150–4.551)
MED22	0.022	0.423(0.203–0.882)
RNH1	0.163	0.647(0.351–1.193)
HM13	<0.001	4.186(2.673–6.555)
EZR	0.344	0.873(0.659–1.156)
PTPRM	<0.001	0.393(0.295–0.525)
MX1	<0.001	1.755(1.477–2.086)
CPSF4	<0.001	5.468(3.375–8.856)
IRF7	<0.001	1.646(1.328–2.039)
MEAF6	<0.001	3.617(1.841–7.104)
RNF130	<0.001	2.735(1.809–4.136)
LMF1	0.014	1.798(1.129–2.864)
ZNF786	0.895	1.043(0.561–1.939)
LIG4	0.630	0.884(0.536–1.458)
BICD1	<0.001	3.603(2.520–5.150)
MAX	0.012	0.351(0.155–0.793)
C5orf45	0.008	0.490(0.289–0.829)
CREM	<0.001	3.270(2.286–4.676)
SUN2	0.009	0.550(0.352–0.859)
NUP62	<0.001	3.530(2.087–5.973)
TPM2	<0.001	1.593(1.299–1.955)
ANKLE2	0.040	3.149(1.055–9.394)
LOXL3	<0.001	2.959(2.101–4.167)
CACNB3	0.263	1.142(0.905–1.441)
MTMR3	<0.001	0.182(0.119–0.277)
RGL1	0.937	0.979(0.572–1.674)
PPIL2	<0.001	0.189(0.100–0.358)
PIAS3	0.003	3.680(1.578–8.586)
RPAIN	0.026	0.482(0.253–0.917)
ANKRD10	0.027	1.564(1.052–2.325)
HAUS1	<0.001	3.503(2.632–4.662)
CADM3	<0.001	0.684(0.574–0.814)
UIMC1	<0.001	4.519(2.071–9.862)
DOCK9	0.035	0.747(0.570–0.980)
NAV2	<0.001	0.452(0.338–0.604)
ATL2	<0.001	4.225(2.112–8.452)
FAH	<0.001	2.598(1.875–3.600)
NHSL1	0.391	0.862(0.613–1.211)
MXRA8	<0.001	2.459(1.928–3.136)
ITGB3	<0.001	1.680(1.460–1.934)
TBX6	0.086	0.773(0.576–1.037)
NAIP	<0.001	1.910(1.391–2.624)
RPLP0	0.764	0.947(0.664–1.351)
ST3GAL6	0.042	0.744(0.560–0.989)
WIPF1	0.116	1.442(0.913–2.278)
FCAR	0.027	1.198(1.021–1.407)
OGG1	0.074	2.145(0.929–4.952)
CCDC141	<0.001	0.611(0.486–0.770)
TEX9	0.243	0.862(0.672–1.106)
ZNF124	0.327	1.220(0.819–1.816)
ZCWPW1	<0.001	0.444(0.308–0.639)
TRAF3IP2	0.006	1.866(1.194–2.917)
CYHR1	0.208	1.347(0.847–2.142)
C1orf61	<0.001	0.722(0.613–0.851)
C11orf80	<0.001	2.235(1.512–3.302)
FAM107B	<0.001	0.492(0.359–0.674)
TECPR1	0.031	0.676(0.474–0.964)
CDC14B	<0.001	0.375(0.214–0.655)
IL11RA	0.148	0.827(0.639–1.070)
NAT9	0.426	0.814(0.490–1.351)
DCTN1	<0.001	0.133(0.071–0.247)
KIF3A	<0.001	0.319(0.244–0.417)
DPEP2	0.005	1.466(1.124–1.913)
APBB3	0.030	0.701(0.509–0.965)
ABCA2	<0.001	0.536(0.406–0.709)
GPD2	0.676	0.865(0.439–1.706)
TCF20	<0.001	0.293(0.205–0.420)
CLASP1	<0.001	0.284(0.183–0.438)
ARMC8	0.033	0.406(0.177–0.931)
ZDHHC16	0.106	0.545(0.262–1.137)
PRKAG2	<0.001	0.522(0.386–0.706)
C22orf29	<0.001	0.389(0.231–0.656)
GIT2	0.010	3.717(1.366–10.113)
CD200	0.025	0.671(0.474–0.951)
DEF8	<0.001	0.178(0.081–0.393)
OAZ1	<0.001	5.103(3.074–8.469)
ANKRD29	0.975	1.003(0.824–1.221)
OGFOD2	0.548	1.191(0.674–2.103)
TSHR	<0.001	0.753(0.679–0.835)
ADCK4	<0.001	2.519(1.620–3.918)
DNAJC14	0.008	4.372(1.481–12.903)
CASZ1	0.686	1.043(0.850–1.281)
ACTA2	<0.001	1.500(1.286–1.748)
ARHGEF7	<0.001	0.396(0.268–0.585)
CADM1	0.009	1.810(1.161–2.820)
TSGA10	0.871	1.032(0.706–1.509)
ZNF524	<0.001	2.018(1.394–2.920)
PCDH1	0.131	0.760(0.533–1.085)
RCAN2	0.213	0.890(0.741–1.069)
BEND7	<0.001	0.303(0.220–0.417)
WDR33	<0.001	0.046(0.015–0.143)
COPS7A	0.009	2.430(1.248–4.733)
MYO5A	<0.001	0.428(0.315–0.581)
PHKB	0.427	1.368(0.631–2.964)
FAM110A	<0.001	0.599(0.446–0.805)
EXTL3	<0.001	4.805(2.907–7.944)
FAM13B	0.117	1.435(0.914–2.253)
CCDC136	0.015	0.720(0.552–0.939)
C11orf74	<0.001	0.218(0.118–0.404)
CFL1	<0.001	4.399(2.310–8.377)

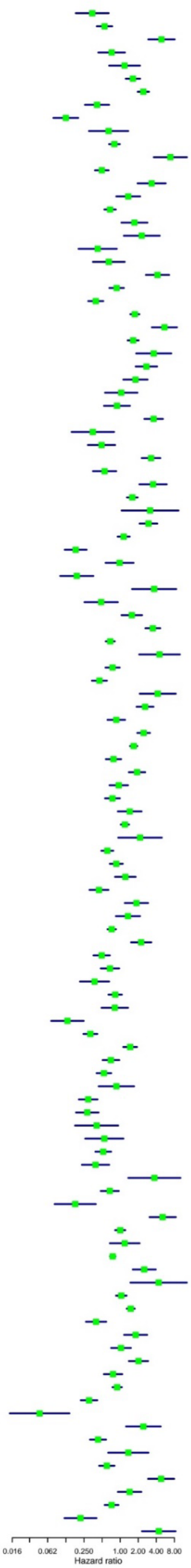

Figure S3 The forest plot for the results of univariate Cox regression analysis in glioma. The results showed that 61 me-sQTL genes identified are high-risk factors.
